# Trypanosome Motion Represents an Adaptation to the Crowded Environment of the Vertebrate Bloodstream

**DOI:** 10.1371/journal.ppat.1003023

**Published:** 2012-11-15

**Authors:** Niko Heddergott, Timothy Krüger, Sujin B. Babu, Ai Wei, Erik Stellamanns, Sravanti Uppaluri, Thomas Pfohl, Holger Stark, Markus Engstler

**Affiliations:** 1 Department of Cell and Developmental Biology, Biocenter, University of Würzburg, Würzburg, Germany; 2 Institute of Theoretical Physics, Technische Universität Berlin, Berlin, Germany; 3 Physics Department, Malaviya National Institute of Technology Jaipur, JLN Marg, Jaipur, Rajasthan, India; 4 Max-Planck-Institute for Dynamics and Self-Organization, Göttingen, Germany; 5 Department of Chemistry, University of Basel, Basel, Switzerland; Washington University School of Medicine, United States of America

## Abstract

Blood is a remarkable habitat: it is highly viscous, contains a dense packaging of cells and perpetually flows at velocities varying over three orders of magnitude. Only few pathogens endure the harsh physical conditions within the vertebrate bloodstream and prosper despite being constantly attacked by host antibodies. African trypanosomes are strictly extracellular blood parasites, which evade the immune response through a system of antigenic variation and incessant motility. How the flagellates actually swim in blood remains to be elucidated. Here, we show that the mode and dynamics of trypanosome locomotion are a trait of life within a crowded environment. Using high-speed fluorescence microscopy and ordered micro-pillar arrays we show that the parasites mode of motility is adapted to the density of cells in blood. Trypanosomes are pulled forward by the planar beat of the single flagellum. Hydrodynamic flow across the asymmetrically shaped cell body translates into its rotational movement. Importantly, the presence of particles with the shape, size and spacing of blood cells is required and sufficient for trypanosomes to reach maximum forward velocity. If the density of obstacles, however, is further increased to resemble collagen networks or tissue spaces, the parasites reverse their flagellar beat and consequently swim backwards, in this way avoiding getting trapped. In the absence of obstacles, this flagellar beat reversal occurs randomly resulting in irregular waveforms and apparent cell tumbling. Thus, the swimming behavior of trypanosomes is a surprising example of micro-adaptation to life at low Reynolds numbers. For a precise physical interpretation, we compare our high-resolution microscopic data to results from a simulation technique that combines the method of multi-particle collision dynamics with a triangulated surface model. The simulation produces a rotating cell body and a helical swimming path, providing a functioning simulation method for a microorganism with a complex swimming strategy.

## Author Summary

African trypanosomes swim incessantly in the bloodstream of their mammalian host. We have asked the question how these parasites actually manage to swim and manoeuver in an environment that is so amazingly crowded by blood cells and that reveals rapidly varying fluid flow speeds that are 50–20.000 times faster than the trypanosome's swimming speed. Our experiments suggest an astute mechanism by which trypanosomes have perfectly adapted to their hostile microenvironment. We found that the pathogens can readily adjust the beating direction of their single flagellum in response to purely mechanical cues. In the blood they exploit the spacing and shape of blood cells for very efficient forward movement that is required for host antibody clearance. When the parasites get trapped, i.e. in the extracellular matrix, they reverse the beating direction and consequently move backwards. The mechanism of flagellar beat switch is unique in nature and represents a genetically fixed trypanosome virulence factor. By introducing innovative technological advances, we have been able to quantify this complex cell behavior with unprecedented spatial and temporal resolution. These include the first numerical simulation of a cell of this complexity, extending the protozoans suitability as a model organism for the regulation of flagellar and ciliary motility.

## Introduction

Blood vessels form a dense network throughout the human body with a total length of about 100,000 kilometers. The vessels diameter ranges from a few micrometers in capillaries to centimeters in the aorta and veins. Blood contains about 45% (v/v) cellular components, which flow with velocities ranging from mm s^−1^ in capillaries to m s^−1^ in the aorta. Viscous forces and laminar flow are dominant in blood circulation. In small capillaries, red blood cells (RBC) move in a single row, while in larger vessels they are thought to accumulate in the channel center due to hydrodynamic flow effects. Despite these fundamental characteristics, blood composition, temperature, pressure and oxygen content differ significantly between vertebrate species. Nevertheless, the parasitic unicellular trypanosomes prosper in the circulation of all vertebrate classes, from fish to bird. Thus, the parasites have evolved by adapting to very different bloodstream conditions.

**Figure 1:**
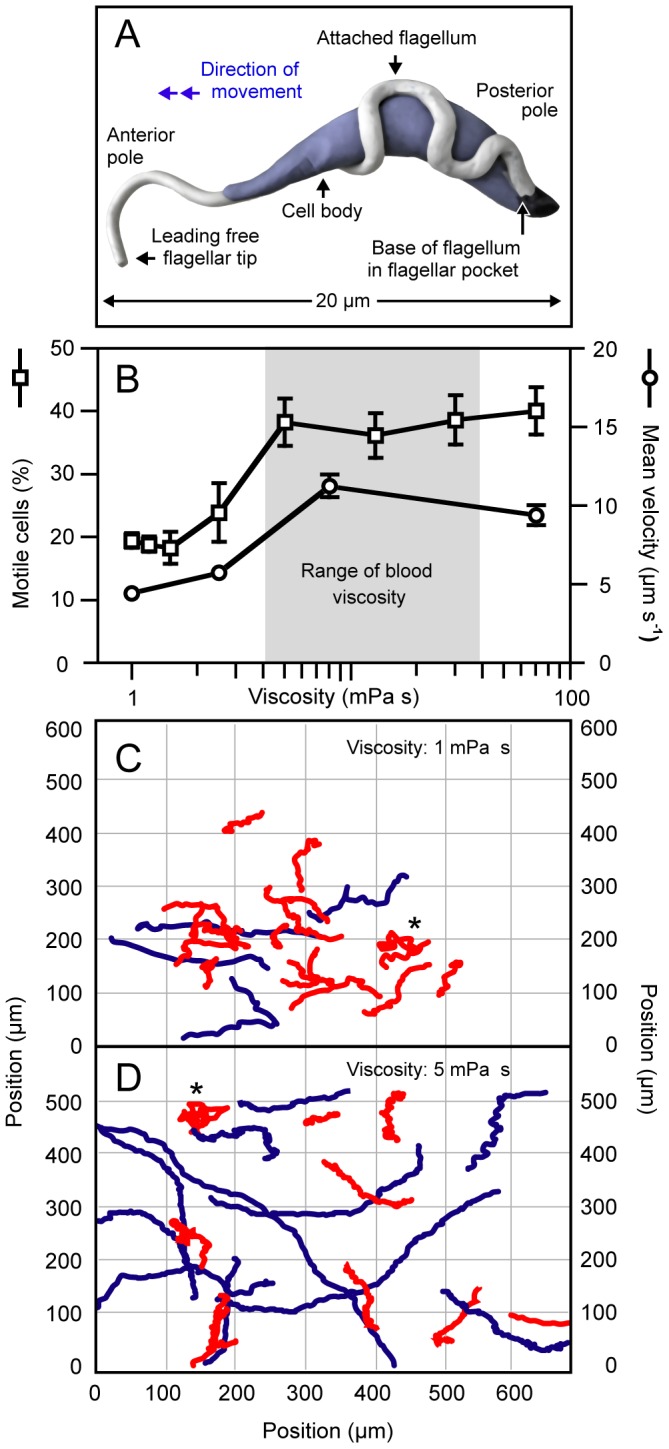
Trypanosome swimming behavior is a function of viscosity. (*A*) Illustration of *Trypanosoma brucei* cell architecture. (*B*) Percentage of motile cells (squares) and mean population velocity (circles) in cell culture medium of varying viscosity, adjusted by the addition of methylcellulose (see Table 1 for concentrations; squares: n = 600–1.000 cells, weighted mean and weighted standard deviation from 3 independent experiments; circles: n>100 cells, mean and standard error). A cell was classified as motile if its trajectory exceeded a length of 150 µm, which corresponds to an average velocity of 5 µm s^−1^ during the 30 seconds of observation. (*C*, *D*) Trajectories of trypanosomes moving for 30 seconds in media with viscosities of 1 mPa s (*C*) and 5 mPa s (*D*). Trajectories showing less than 150 µm of directional motion are indicated in red and correspond to non-persistently swimming cells. Cells marked with an asterisk were in the final stage of cell division, having two opposing flagella and exhibited tumbling paths characteristic for this stage.

Some trypanosome species cause deadly diseases in livestock and man, e.g. the African sleeping sickness. Human African Trypanosomiasis (HAT) is an exemplary disease of poverty. There are only very few and rather ancient drugs available, which in addition are highly toxic. Most critically, in many sub-Saharan countries, health agencies have essentially lost control of HAT due to social and geopolitical problems; consequential poor public health implementation has resulted in widespread emergence of drug resistance. Thus, new medication is urgently needed. Unraveling the unique cellular and molecular features that distinguish trypanosomes from other eukaryotes has been a prime goal in the search for promising drug targets, however, success has been limited so far. An alternative approach appears to be to study the behavior of trypanosomes in their natural environment, namely the mammalian bloodstream, where the cells are constantly opsonized with antibodies and serum factors. The only barrier that shields trypanosomes from the host is an astonishingly dense cell surface coat, which is made of 10^7^ copies of the same type of lipid-anchored, variant surface glycoprotein (VSG). The trypanosomes use a system of antigenic variation to evade the host's immune response, whereby they randomly switch the exposed variant glycoproteins (VSG) and thus escape detection [1]. Due to the large number of structurally related but immunologically distinct VSGs, vaccination against HAT appears impossible. However, a second immune evasion strategy could prove more versatile for intervention as it directly involves cellular motility, which is thought to be essential for the parasites [2]–[4]. Bloodstream form (BSF) trypanosomes swim to rapidly remove surface-bound antibodies [5], [6]. For propulsion, they utilize a single leading flagellum, which emerges from the flagellar pocket, follows the cell body, to which it is attached, and protrudes freely at the anterior of the cell (Fig. 1*A*). The motion relative to the surrounding fluid generates a hydrodynamic drag, which causes antibody-bound VSGs to drift in the plane of the plasma membrane towards the trailing posterior end and into the flagellar pocket [5]. This invagination of the plasma membrane is the only place where endocytosis, an extremely fast process in trypanosomes, takes place [7]. The mechanism of hydrodynamic protein sorting greatly accelerates the diffusion-limited uptake of host antibodies, provided that trypanosomes exhibit fast directional motion. The aim of the present study was to elucidate in detail the biomechanics of trypanosome motility and relate this to the parasites life in blood.

## Results

### Environmental influence on swimming behavior

The swimming behavior of cultivated trypanosomes appears to be highly variable, with few cells showing persistent directional motion (Table 1). Persistence is defined here by continuous directional movement for at least 150 micrometers, whereas non-persistent motion is characterized by shorter swimming trajectories interrupted by tumbling phases, during which the trypanosome stops translocation and changes its orientation [8], [9]. In cell culture, the measured mean population velocity was relatively slow ((5.7±0.11) µm s^−1^; SEM, n = 979). This is slower than the velocity required for efficient removal of host antibodies [5]. In contrast, in blood the cells reached much higher velocities of 30 µm s^−1^. Obviously, the physicochemical conditions in blood and cell culture differ greatly. Therefore, we systematically analyzed the motility of trypanosomes in varying environments. Possible factors that influence swimming behavior include chemical cues, oxygen content, pressure, viscosity, flow, confinement and presence of blood cells.

**Table 1:** Viscosity of the medium affects motility of trypanosomes.

	Viscosity (mPa s)	Persistently swimming cells (%)*
Blood, infected	4.5	90
Blood, non-infected	4.5	86±9
Blood, non-infected diluted 1∶4	N.D.	55
Blood Serum	1.20	31±12
50% Serum	1.05	30±4
HMI-9	0.95	26±4
HMI-9+0.2% Methylcellulose	2.5	24±5
HMI-9+0.4% Methylcellulose	5.2	38±4
HMI-9+0.6% Methylcellulose	24.3	36±4

* For each experiment, at least 600 cells were analyzed.

In blood freshly drawn from infected mice, virtually all cells swam persistently (Table 1). In contrast, less than a third of cells were persistent swimmers in standard cell culture medium and velocities varied significantly. We found no difference in the swimming behavior of trypanosomes in blood of pre-infected and uninfected animals, ruling out that the parasites secrete a motility-promoting factor. Blood plasma or serum only marginally influenced the percentage of mobile cells (Table 1). Thus, chemical cues are unlikely to affect trypanosome motility in the bloodstream. We also observed the reduction of oxygen partial pressure to have no influence on motility. However, when we raised the viscosity of the cell culture medium, the number of persistent swimmers increased, as well as the velocity of trypanosomes (Fig. 1*B*). Upon addition of 0.4% (w/v) methylcellulose the viscosity of the medium equals that of blood. Under these conditions the percentage of persistently swimming cells doubled and the mean population velocity almost tripled (Fig. 1*B*–*D*).

### Flagellar force

Trypanosomes were capable swimmers even when viscosity was raised to 4000 mPa s, three orders of magnitude higher than that of blood. This compares to mammalian spermatozoa, which are also adapted to move in a broad viscosity range. In sperm, the flagellar beat frequency is decreased in high viscosity medium, but the progressive velocity does not change, as kinetic efficiency rises [10], [11]. This probably involves the interaction with microstructures contained in the methylcellulose solution [11]. The forces trypanosomes would need to exert in order to move through homogenous fluids of such high viscosity are significant. We assume that the increase in velocity, without the need to produce such high forces, can be explained by methylcellulose forming loose and quasi-rigid networks consisting of long, linear polymer molecules. This was suggested first by Berg and Turner in 1979 for bacteria, cilia and flagella [12] and subsequently mathematically developed for certain bacteria [13]. As these bacteria do, small organisms like trypanosomes, even though 3 to 4 times larger in diameter, also seem to be able to wriggle through these networks without experiencing the resistive force of the apparent macroscopic viscosity.

We have estimated the minimal force that the flagellum generates to be 5 pN by bead displacement experiments (Video S1). We also found that trypanosomes produced a force of maximally 100 pN, as they were not able to bend the various poly-dimethyl siloxane (PDMS)-pillars used in our experiments. The force required for bending these pillars is similar to that needed for the deformation of RBC. This agrees with the fact that although RBC can be readily displaced by trypanosomes, erythrocytes cannot be deformed by the parasite (Video S1). Thus, the force exerted by the flagellum is at least one order of magnitude lower than reported previously [14]; the most likely numbers have recently been measured by optical trapping experiments and are below 10 pN [15]. In fact, for swimming, trypanosomes do not require larger forces; the absolute value of the propulsive force that has to be generated equals the viscous drag force acting in the opposite direction. The drag force F can be calculated by the Stokes equation, assuming the cell body to be a sphere with known radius:
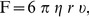
(1)where η is the dynamic viscosity of the medium, *r* is the radius of the sphere and υ is the swimming velocity. For a trypanosome (r = 1.5 µm) swimming in cell culture medium (η = 0.95 mPa s) at a velocity υ of 20 µm s^−1^, the viscous drag force is 0.54 pN.

### Efficient directional movement in pillar arrays simulating blood cells

**Figure 2:**
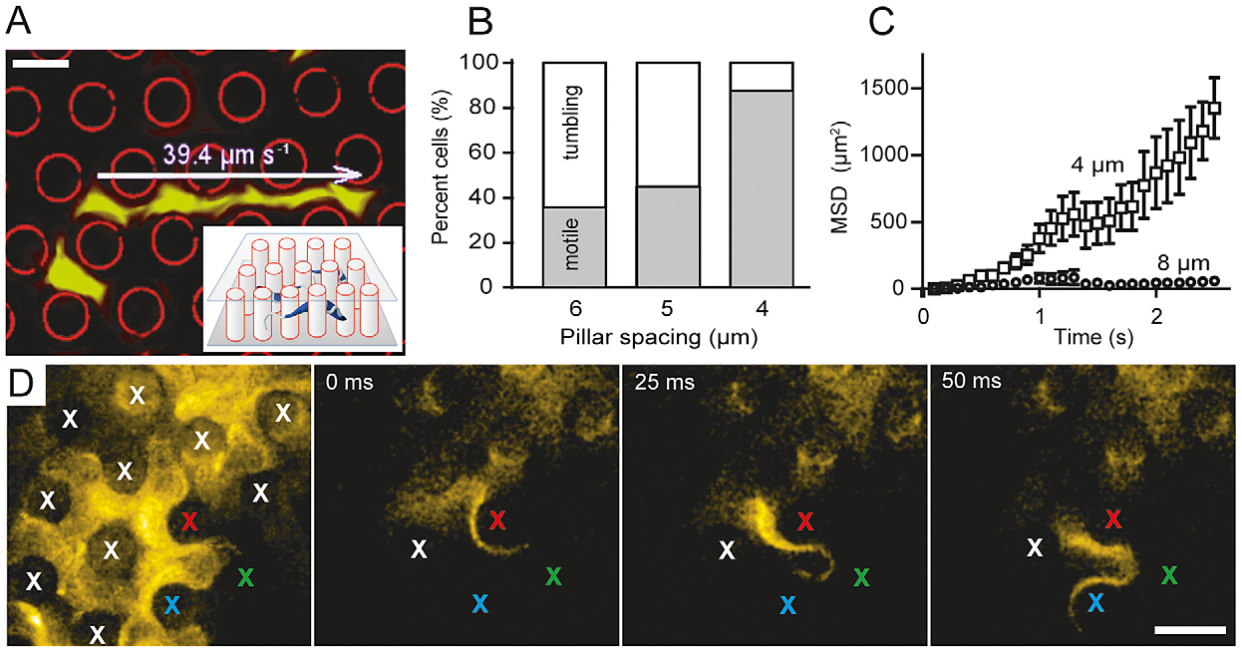
Efficient trypanosome motility requires mechanical interaction with obstacles similar to red blood cells. (*A*) Time-projection of a fluorescently labeled trypanosome (green) moving in a regular pillar array with 4 µm spacing. The fluorescence projection image was merged with a bright-field image of pillars, which had been transformed to a binary image and false-colored red. Within these 4 µm-pillar arrays cells reached their maximum swimming velocity of about 40 µm s^−1^. Inset: Illustration of the experimental setup. (*B*) The pillar spacing determines the percentage of persistently swimming cells. Almost 90% of trypanosomes were classified as motile cells in arrays with 4 µm pillar distance, which corresponds to the mean spacing of erythrocytes in blood (n = 150). Wider spacing led to a sharp decrease in directional motility. (*C*) The dynamics of the cells net directional movement as represented by the mean squared displacement (MSD) plotted over the observation time points to a fast, super-diffusive swimming behavior in pillar arrays of 4 µm spacing. Trypanosomes propagate far more effectively in 4 µm-spaced pillar arrays than in wider spaces. (n = 12; mean and SEM). (*D*) Maximum intensity projection (left) and selected still images from a high-speed fluorescence (xyt) image series of a fluorescently labeled trypanosome swimming through an array of pillars (4 µm spacing), acquired at a frame-rate of 400 images per second (Video S7). Note that the curvatures of the flagellum and the cell body closely reflect the shape of the pillars (positions indicated by ‘x’). Scale bar = 5 µm.

The viscosity of blood directly depends on the concentration of cellular components [16], [17] and the presence of particles renders blood a non-Newtonian fluid, meaning that its viscosity changes with flow speed. This fact, in addition to continuous, vigorous self-mixing and formation of erythrocyte stacks (‘rouleaux’), makes it difficult to measure cell motility in blood directly [18]. Hence, we aimed to quantify the behavior of trypanosomes in environments that resemble aspects of blood physics, but are defined and more easily manipulable. Initially, we chose suspensions of different kinds of particles that produce viscous fluids similar to blood. We generally observed enhanced cell motilities in the presence of particles, independent of their size, shape and mass. Live and chemically fixed erythrocytes influenced trypanosome motion in a manner similar to polystyrene and metal beads or nanodiamonds (Video S1). However, blood is a self-mixing system, meaning that the position of cells is continuously randomized due to hydrodynamic flow under confinement. In order to simulate this complex situation we used continuous flow microfluidics, which to date however, is not compatible with high-resolution, quantitative microscopic imaging of fast moving objects in three dimensions. Therefore, we devised a scenario that resembles a “frozen” suspension. Trypanosome motility was measured in arrays of regularly aligned inert PDMS pillars in order to simulate the crowded environment of the bloodstream (Fig. 2*A*). We observed a striking increase in the percentage of persistently swimming cells and higher velocities for cells mechanically interacting with pillars (Fig. 2*B*). About 90% of trypanosomes exhibited a fast and directional motion in arrays of pillars whose size (8 µm) and regular spacing (4 µm) was comparable to RBC in blood (Fig. 2*C, D*). A maximum speed of 40 µm s^−1^ was measured within these arrays, which is almost 8-times faster than the mean population velocity in cell culture. No difference was found when the experiments were conducted in trypanosome dilution buffer (TDB) instead of culture medium, ruling out that compounds from fetal calf serum were involved. We conclude that objects with the size and spacing of RBC are required and sufficient to promote maximum directional forward velocity of trypanosomes (Table 2) and consequently, the effectual removal of cell-surface bound host antibodies.

**Table 2:** Maximum swimming velocity of trypanosomes in different media.

Medium	Maximum velocity (µm s^−1^)
HMI-9	21
HMI-9+Collagen (0.01 mg/ml)	23
HMI-9+Methylcellulose (0.5% w/v)	32
PDMS-Pillars	39
Bovine blood	35

Noteworthy, in the presence of narrower spaced pillar arrays, as well as in artificial collagen networks, around half of the persistent swimmers were observed to move backwards, i.e. with the flagellum trailing. This behavior had never been observed for wild type cells, but only for motility mutants with the outer dynein arms of the axoneme missing [5], [19]. It was tempting to speculate that pure mechanic resistance in close-meshed environments could initiate a simple but faultless trap escape mechanism by triggering trypanosome backward motion.

### The mechanism of motility deciphered by high-speed imaging

In order to understand the complex swimming behavior, we detailed the mechanism of movement for trypanosomes swimming either forwards, backwards or tumbling.

**Figure 3:**
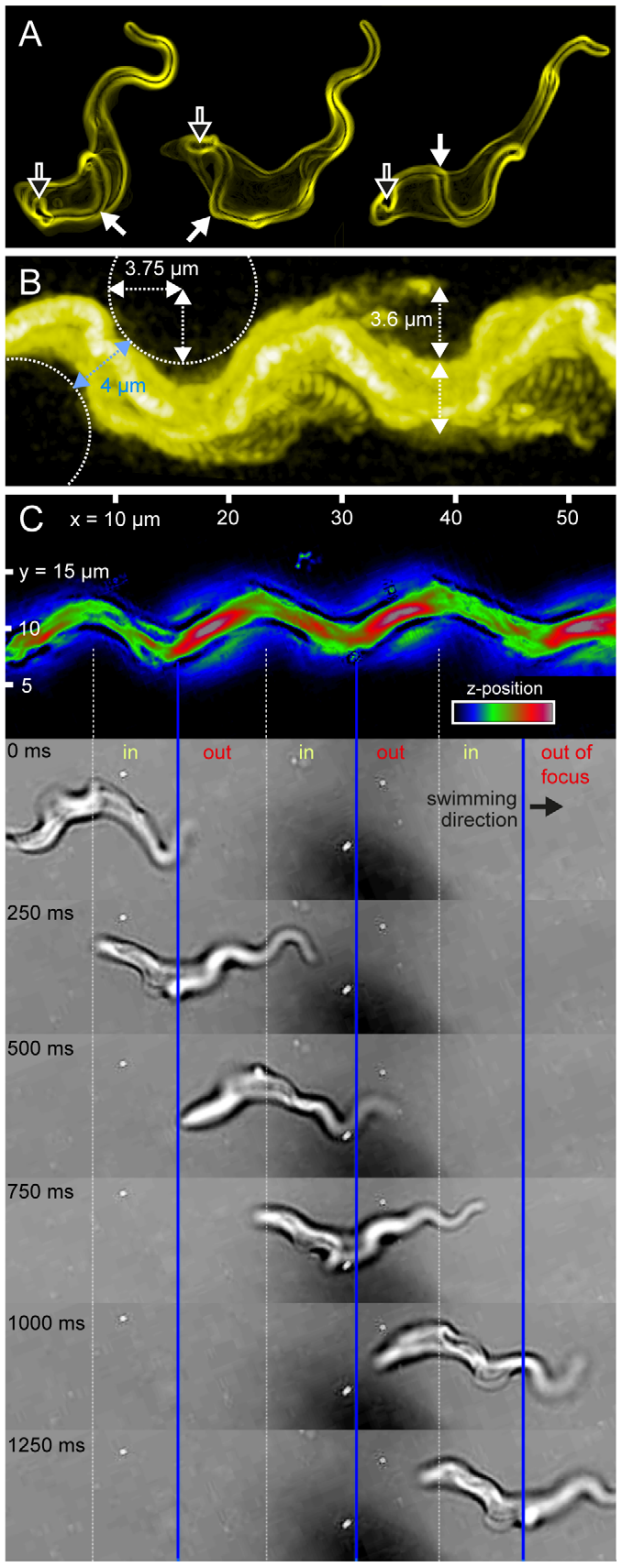
Trypanosome mode of motility is a consequence of the architecture of the cell, which resembles an adaption to life in blood. (*A*) Volume-rendered models of trypanosomes, surface-labeled with AMCA-sulfo-NHS. The course of the flagellum attached to the cell body (closed arrows) is clearly visible from the flagellar pocket (open arrows) to the anterior free end. The flagellum characteristically describes a turn of about 180 degrees around the cell body, counter-clockwise in swimming direction. This can unequivocally be seen in the animated three-dimensional view of the volume models (Videos S2, S3). The cells are representative examples of directionally swimming trypanosomes, as was confirmed by matching the 2D-views of the model with the single images from fluorescence high speed videos (Fig. 3*B* and Video S4). (*B*) Time-projection (xyt) of a directionally swimming cell fluorescently labeled with Atto488-NHS and recorded at 400 fps (Video S4). The projection reveals a wave pattern with a frequency of 2–3 Hz and amplitude of 3.6±0.2 µm. This amplitude matches the radius of erythrocytes as illustrated by dashed circles. The width of the swimming trypanosomes trajectory is close to 4 µm, which is not only optimal for efficient motion in pillar arrays (Fig. 2), but also corresponds to the mean distance of erythrocytes in blood. Note that no pillars were present in this experiment. Thus, the appearance of the characteristic swimming trail of trypanosomes is not a consequence of the presence of obstacles, but a product of cell shape and mode of motility and hence it is genetically fixed. (*C*) Out-of-focus information supports the view of a helical type of trypanosome movement. Top: False-colored time projection of a bright field image series, recorded at 500 fps (Video S8). The average intensity in the bright field images corresponds to the z-position of the respective parts of the cell body. In this case, the parts of the body that are out-of-focus while swimming through the field of view are imaged with brighter intensity. This information is quantified in the false colored xyt-projection. Following the trypanosomes path, high intensities (red) and lower intensities (green) periodically alternate, corresponding to out of focus and in focus regions respectively, as seen in the single images of the time series (below). Taken together, the directionally swimming cells describe characteristic wave patterns along all three spatial axes. Considering the observed pattern of flagellum movement relative to the cell body, these results strongly support a rotational motion of persistently swimming trypanosomes around their anterior-posterior axis, which represents an adaptation to life in blood.

High-speed transmitted light microscopy imaging allows analysis of cell motility with sufficient resolution in space and time [20]. Here, we introduce fluorescence microscopy utilizing sCMOS technology, which combines kHz frame rates with very high sensitivity. High-speed fluorescence microscopy has the dual advantage of providing defined out-of-focus information as well as high-resolution structural content, allowing the derivation of three-dimensional information at unprecedented temporal resolution. Using fluorescence surface labeling and high speed imaging, we observed the exact course of the flagellum and the cell body around individual pillars during successive flagellar beats, unequivocally demonstrating the adaptation of cell morphology to the surrounding erythrocyte-sized obstacles (Fig. 2*D*). Only forward swimming cells whose flagellar wavelength and amplitude, as well as the resulting dynamic curvature of the cell body matched the spacing between PDMS-pillars achieved maximum directional velocity. These cells exhibited continuous tip-to-base beats of the flagellum at a regular frequency of (18.3±2.5 Hz). The amplitude of the free flagellar tip, defined as half the maximum transverse displacement of the flagellum relative to the anterior-posterior cell axis, was (3.3±0.7 µm). This specifically allowed mechanical interaction with correspondingly narrow-spaced objects. Additionally, the moving cell body described a helical path of similar amplitude (3.6±0.2 µm), even without obstacles being present (Fig. 3B), revealing that hydrodynamic drag alone translates into rotation of the entire cell. In fact, the trypanosomes exploited the mechanical resistance of pillars periodically acting on opposite sides of the cells for efficient locomotion, The rotation of the parasites allowed interaction of the flagellum with the environment equally in all three dimensions, thereby maximizing the probing space of the flagellar tip (M. Engstler et al., unpublished).

### The morphology of the cell body causes its rotational movement

**Figure 4:**
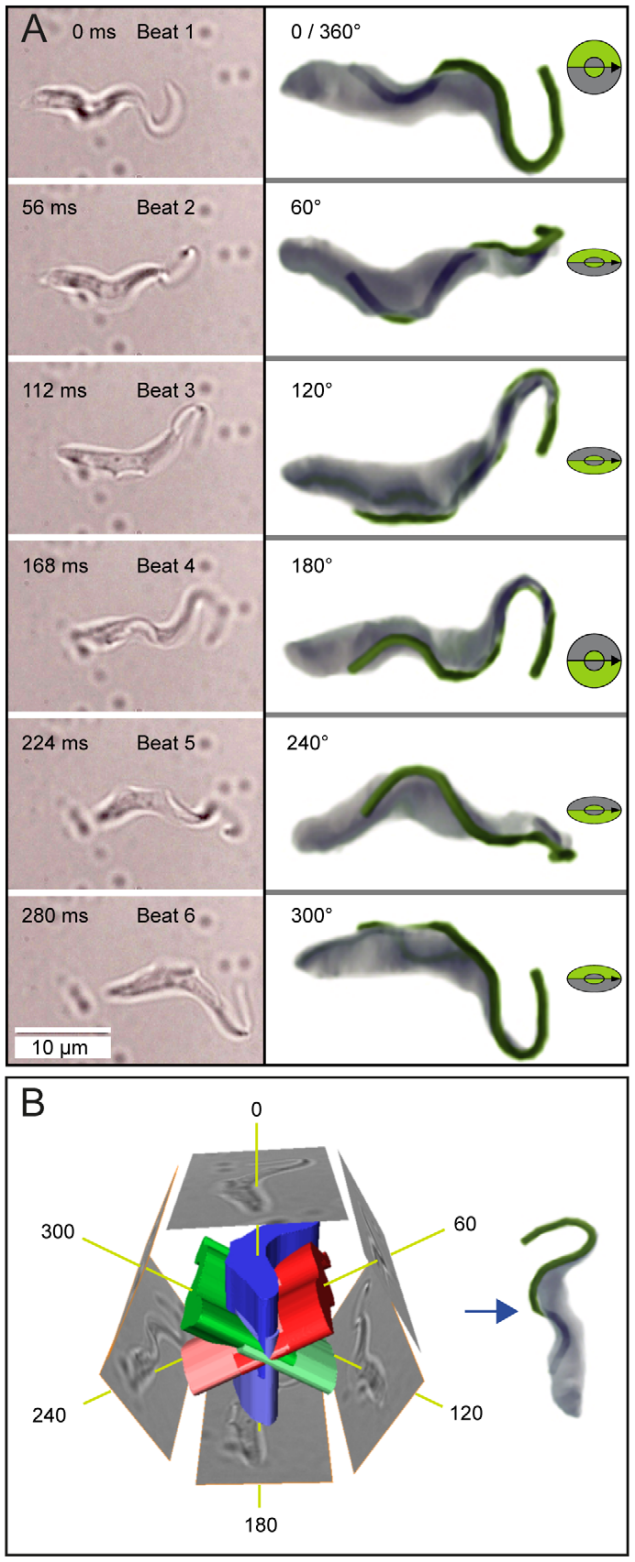
Time-dependent tomography proves the rotation of the cell body during locomotion. (*A*) A bright-field image series of a directionally swimming trypanosome was acquired at 500 fps. Successive flagellar beats were analyzed and one image depicting the beginning of each beat was selected. This was every 28^th^ frame, corresponding to a beat frequency of 18 Hz (left). After six beats the cell periodically reached the same spatial orientation. The edges of the cell were traced in the two-dimensional images and extruded to create three-dimensional objects. The six resulting 3D-contours were aligned on one anterior-posterior axis. The models were successively rotated around this axis in 60° steps per beat. The intersecting regions of the rotated objects were calculated and extracted. This produced a three-dimensional volume object that closely resembled a trypanosome cell body. When rotated by 60° per step the resulting views of the three-dimensional body compared well with the original microscopic images (right). The flagellum was traced and modeled manually, as the flexible, free anterior part cannot be aligned together with the cell. The flagellum (green) is shown superimposed on and rotated together with the cell body (right). This “time-dependent“ tomography method only produced a recognizable trypanosome model assuming 60°±5° turns per beat. All analyzed cells produced valid models in the range of 50°±10°. Note that these results formally prove a rotational mode of trypanosome motion. (*B*) Schematic illustration of the principle of time-dependent tomography.

It is the asymmetry of the cell body that causes trypanosomes to rotate. From its origin at the flagellar pocket to the anterior pole of the cell, the flagellum wraps around the cell body in a turn of approximately 180 degrees (Fig. 3*A*) (Videos S2, S3). This turn is completed after a flagellar course of 11 µm on average (Video S2). The remaining process of the flagellum towards the anterior pole of the cell follows the convex side of the cell surface without wrapping around the body significantly. This means that the helical chirality of the trypanosome is based on a flagellum that makes less than one complete turn around the cell; so the trypanosome (trypanon [gr.] = auger) itself does not resemble a corkscrew, but its mode of motion does (Fig. 3*B,C*). The rotation becomes evident through high-speed fluorescence microscopy analysis. Tracking the relative position of the flagellum along the cell body reveals that it always entered the focal plane on the right side of the cell, respective to the direction of movement, and left it on the left side (Video S4). Furthermore, by time-dependent tomography, 3D-representations of cells were successfully calculated from high-speed time-lapse series. This novel approach can produce a valid 3-dimensional model of a given body only if this object rotates unidirectionally with constant angular velocity. For a trypanosome the angular rotation was determined to be (50±10) degrees per flagellar beat, proving that the parasites exhibit a rotating type of motion (Fig. 4). These results are perfectly consistent with the measured beat frequency of (18.3±2.5) Hz (n = 60), and a mean rotational frequency of (2.8±0.4) Hz (n = 58). This corresponds to (8±2) single flagellar beats for a full rotation. Video S5 shows representative high-speed data sets selected from several hundred xyt-image series.

### Forward propulsion by tip-to-base flagellar beats

**Figure 5:**
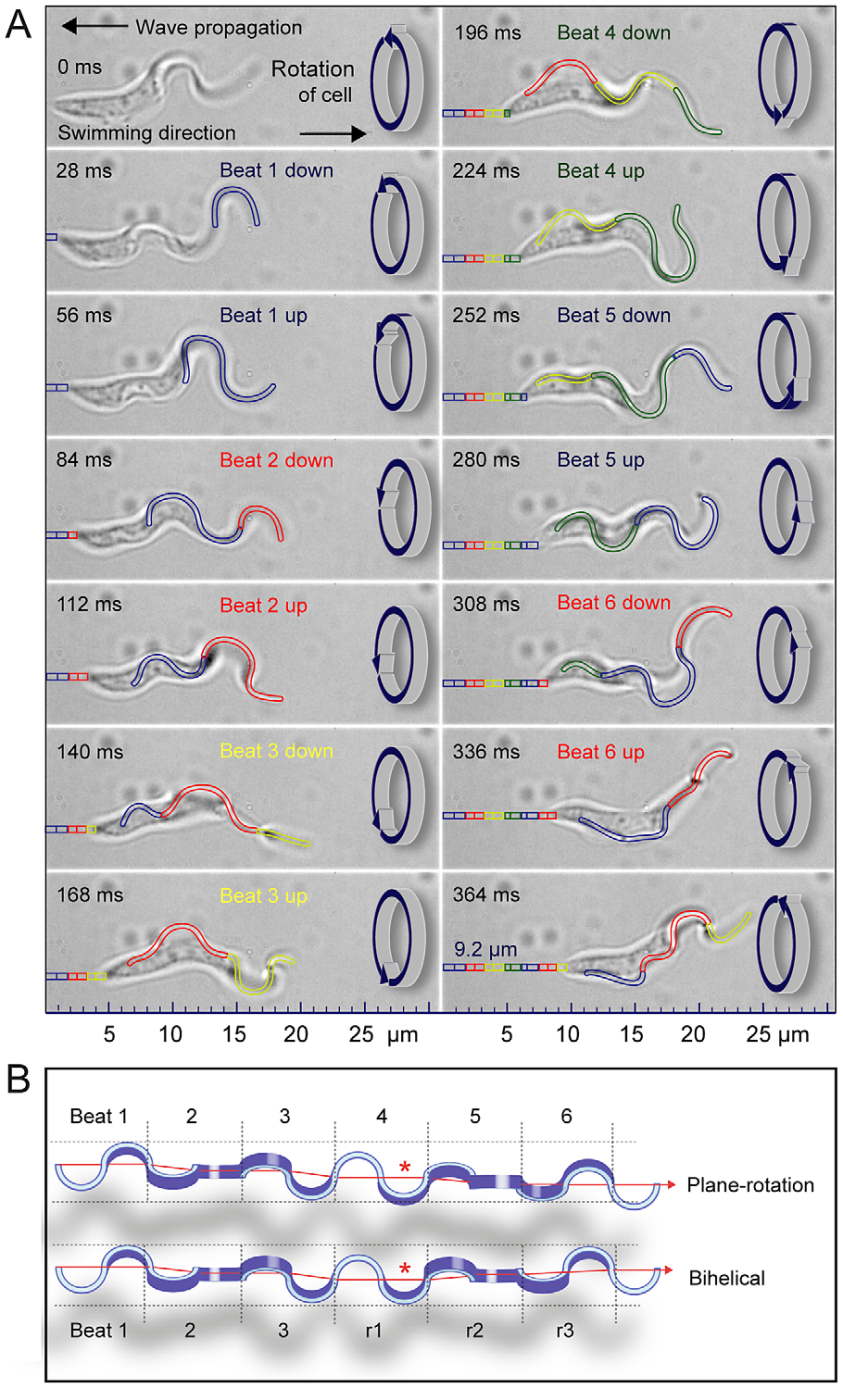
The plane-rotational motion of directionally swimming trypanosomes. (*A*) Bright-field images from a xyt-series acquired with 500 fps (Video S8). Each image shows the beginning of successive up- or down-strokes of the flagellar tip, meaning a new wave starts at the anterior end of the flagellum every two images, i.e. after 28 frames. At each point in time, 2–3 wave crests are visible progressing from the anterior (tip of flagellum) towards the posterior part of the swimming cell. Every single up- and down-stroke causes forward motion in the opposite direction of wave propagation, seen as discrete translocations of about 0.7 µm, due to the helical path of the body and rotation of the cell body by about 25 degrees counter-clockwise. The cell was swimming at a speed of 25 µm s^−1^ and travelled 9.2 µm in 364 ms. In this period the flagellum produced 6.5 beats. (*B*) Illustration of the plane-rotational mode of motion compared to a bihelical mode (14). The latter requires a reversal of the rotational motion of the cell body after a turn of 180 degrees (marked by red asterisk), while the plane-rotational motion results in a continuous rotation in the same direction. Although the overall impression of these models appears rather similar, the physics underlying these two types of motion are fundamentally different.

For persistently moving cells, the trypanosome flagellum produced waves that moved unidirectional from the flagellar tip to the base with a constant frequency of about 18 Hz. Bihelical flagellar waves were not observed [14]. While the amplitude was increasingly damped by the cell body, a postulated change in the frequency of the flagellar beat [21] could not be confirmed (Fig. 5). Due to the virtual absence of inertia at very low Reynolds numbers [22], [23], every single flagellar beat produced a distinct and immediate propulsive force. The resulting locomotion could be visualized in discrete steps, due to the simultaneous rotation of the cell body and the therefore helical path around the axis of movement (Fig. 5*A*, Video S8). The unidirectional rotation of the flagellar beat plane is illustrated in Fig. 5B, in comparison to a theoretical bihelical mode [14]. Although the overall impression of these models appears rather similar, the physics underlying these two types of motion are fundamentally different.

The major propelling force of trypanosome movement is produced by the beat of the free anterior part of the flagellum. This force in the anterior direction, together with the hydrodynamic drag force consequently produced in the opposite direction, causes rotation of the posterior flagellar 180° left-hand turn and accordingly of the attached cell body. In this context it may be worth mentioning that we did not find any support for the existence of an undulating membrane, which is generally thought to drive trypanosome motion. For various reasons the existence of such a flexible, fin-like extension between cell body and the attached flagellum in *T. brucei* may be doubted. Firstly, to the best of our knowledge neither the literature nor our own experiments (see for example Fig. 3*A* and Videos S2, S3) provide any compelling microscopic evidence for the presence of an undulating membrane. Secondly, the physical tethering of the paraflagellar rod to parts of the subpellicular cytoskeleton renders the connection between flagellum and cell body inflexible to the extent of contrasting with the concept of an undulating membrane driving trypanosome motion.

### Persistent backward swimming by reversal of flagellar beats

After having detailed the movement of continuously forward swimming cells, we examined trypanosomes not being persistently propelled by tip-to-base beats. In micropillar arrays, about half of swimming trypanosomes were observed to move backwards by effectively reversing their flagellar beat direction (Video S11). The unique switch to base-to-tip beats is characteristic of trypanosomes and has been postulated to accompany tumbling phases and reorientation of the cell body in cell culture [19], [20]. Here, for the first time, we show that continuous base-to-tip beats result in persistent backward movement.

The base-to-tip beating trypanosomes were translocated in the posterior direction with each beat and the cells rotated counter-clockwise, viewed in the direction of movement (Video S11). This means the flagellar propulsion and the consequent rotation of the cell body observed in forward movement were reversed in base-to-tip beating cells. The frequency for consecutive base-to-tip beats in backward moving cells (13.1±0.8 Hz) was lower than for forward swimming parasites (18.3±2.5 Hz). The base-to-tip beats typically produced a more irregular wave pattern with frequently higher amplitudes than tip-to-base beats, enabling the flagellum to fold back against itself, producing a hook-like waveform instead of a regular sine-wave. Therefore, backwards swimming trypanosomes followed paths of variable amplitudes, dependent on the constraints that the viscous surrounding or the presence of micropillars presented.

### Viscosity determines persistence of directional movement

Persistent backward motion was only observed with cells swimming in collagen networks, in methylcellulose or between narrow-spaced pillar arrays (Video S11). Under conditions offering no confinement and hence no mechanical resistance, continuous base-to-tip beating was not sustained. Instead, base-to-tip and tip-to-base beats permanently alternated. This resulted in very short intervals of for- or backward motion interrupted by beat reversal. While beats of changing direction were initiated, the resulting flagellar waves were simultaneously propagated and thereby generated superimposed waveforms. Thus, the appearance of a so-called bihelical mode of trypanosome motion [14] in fact reflects transition periods, that do not contribute to, but, quite the contrary, interrupt directional motion. Importantly, the occurrence of base-to-tip beats was observed to result in rotational movement perpendicular to the anterior-posterior cell axis. In this way, even very few base-to tip beats interrupting the continuous tip-to-base beating of forward moving cells will alter the trypanosomes swimming direction (Video S12). In cell culture, i.e. in the absence of confinement, frequent reversals of the flagellar beat direction caused successive directional changes that led to the characteristic, albeit artificial tumbling movement of trypanosomes (Video S13).

### Numerical simulation of trypanosome movement

**Figure 6:**
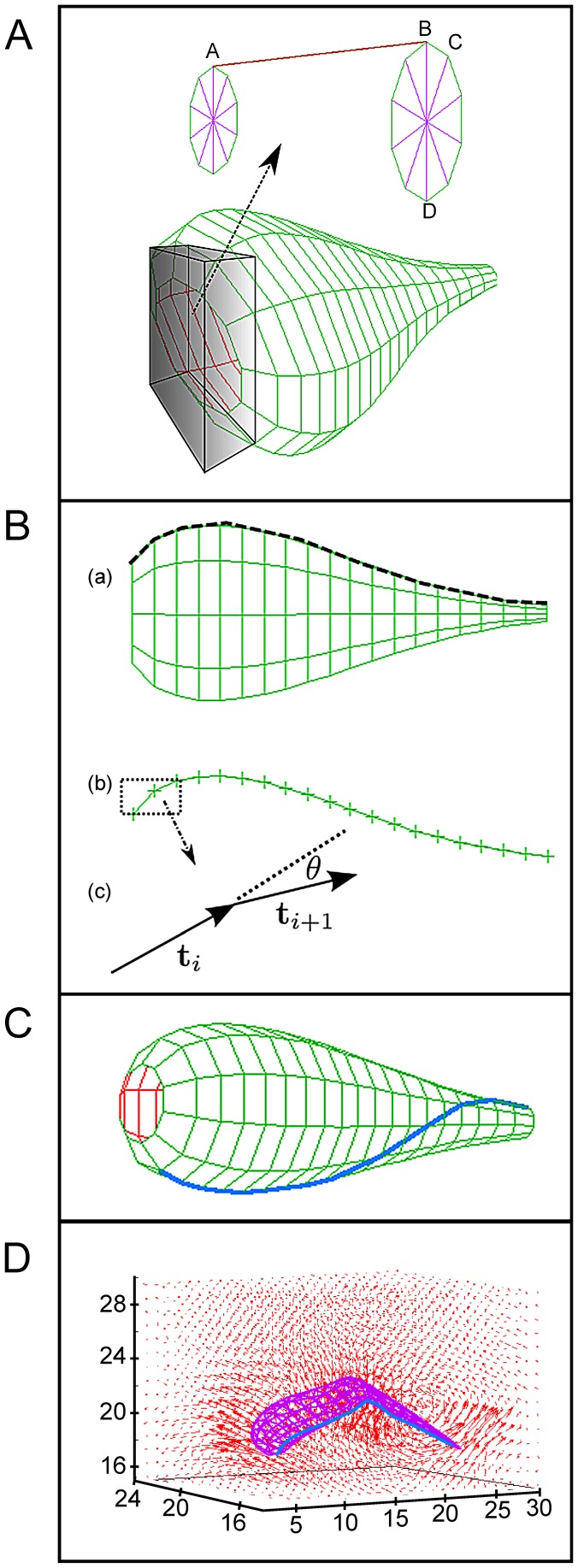
A triangulated surface model of an African trypanosome. *(A)* The elongated cell body is modeled through a net of vertices connected by springs and an additional bending rigidity (for details see Materials and Methods section). (*B*) (a) Side view of the trypanosome model. (b) The dotted line shown in (a) discretized into vertices along which the bending potential is applied, (c) shows how the tangent vectors t*_i_*, t*_i_*_+1_ and the angle *θ* are defined using the discretized vertices. (*C*) The trypanosome surface model at the beginning of the simulation with the helical flagellum indicated as the blue colored line. (*D*) Average flow field of the model trypanosome swimming as obtained from simulation with multi-particle collision dynamics.

**Figure 7:**
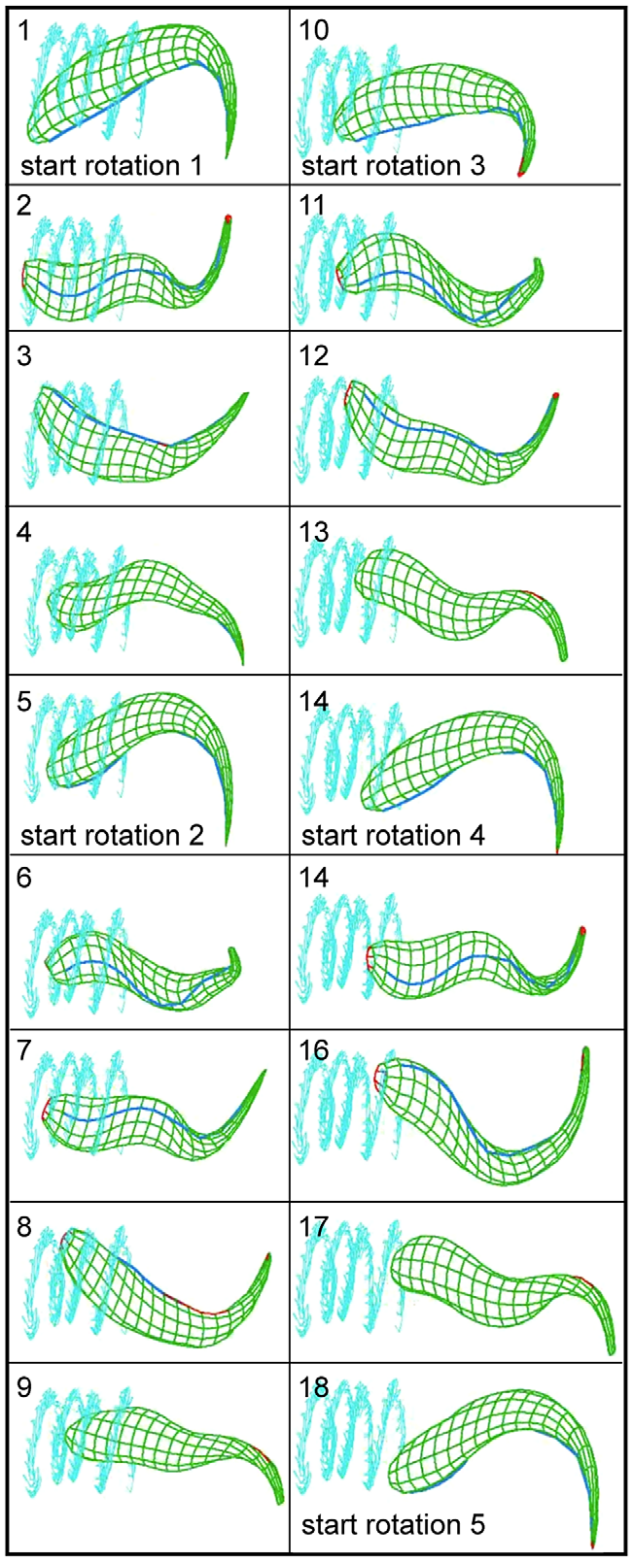
The simulated motion of the trypanosome model using multi-particle collision dynamics confirms the experimentally observed mode of motion. Panels 1–18 show selected frames of an exemplary simulation (see Video S13). The pictures illustrate four full cell rotations. The path of the posterior pole of the cell is shown in cyan color. Due to rotation of the cell body the simulated flagellum (blue color) always appears and disappears on opposite sides of the cell.

Many of our conclusions are based on the careful interpretation of high-resolution microscopic data, which is a complex procedure, given that it involves deducing 3-dimensional information from 2-dimensional image series. Therefore, we corroborated our experimental data with a simulation technique combining the recently developed method of multi-particle collision dynamics to simulate viscous fluid flow [24]–[28] with a triangulated surface model [29]. The cell body surface was shaped in such a way, that bending potentials could be applied along the long axis of the body, in order to simulate the stiffness of the trypanosomes cytoskeleton (Fig. 6*A, B*). A flagellum was defined and a sine wave running from tip to base applied to it, exactly according to our experimental 3d-microscopy data (Fig. 6*C*). The flow field created by the cell propelled the body effectively in the opposite direction to the flagellar wave. The simulation resulted in the rotation of the whole cell body and in a helical swimming path as observed in our experiments (Fig. 7, Video S14). Furthermore, when a sine wave was applied to the flagellum running in the opposite direction (base to tip), the simulated cell body rotated around axes perpendicular to the anterior-posterior axis (Video S14), exactly as observed above for trypanosomes experiencing intermittent flagellar beat reversals. Without mechanical interaction with the surrounding viscous fluid, the model cell body in the numerical simulations neither translocated along nor rotated around the anterior-posterior axis. This behavior confirms that the course of the flagellum along the cell body as well as the direction of flagellar waves are responsible for the basic steps of trypanosome motility, which are modulated *in vivo* by the physical micro-environment to produce the apparent complex swimming mode.

### The mechanism of motility is adapted to the movement between blood cells

Having established that the trypanosome's mode of motility is a consequence of its microenvironment leaves us with the question as to whether it is a genetically fixed trait. We have shown that inversion of swimming direction and flagellar beat reversal can be triggered by knock-down of a single gene product (DNAI1) ([5]; M. Engstler et al., unpublished). For efficient interaction with the microenvironment, however, any gene product contributing to cell shape and stiffness should be considered, which renders a precise genetic analysis difficult. However, there is one last observation that might pave the way for a genetic screen. High-speed analyses clearly showed that all forward swimming cells displayed exactly the same type of rotational movement, in the absence or presence of obstacles (Fig. 3*B*, Videos S4, S5, S6, S7, S8, S9, S10). The amplitude and frequency of flagellar waves are identical for cells swimming in microstructures, in blood or in cell culture medium. However, the optimal transduction of force into speed is dependent on a three-dimensional array of obstacles. This means that the mechanical adaptation to the physical topology of the microenvironment could well be genetically fixed, enabling the cells to move with maximal velocities in their natural habitat. In fact, for trypanosomes the selective pressure to swim faster than in cell culture medium is evident; it is only at velocities greater than 20 µm s^−1^ that the hydrodynamic removal of host antibodies becomes effective.

## Discussion

The physical forces acting in and around cells are moving into the focus of attention, not only of biologists, but also of physicists and engineers. Hydrodynamic flow impacts on cell surfaces even at the scale of molecules [5]. A mechanism exploiting these forces is critical for the survival of a deadly parasite, the African trypanosome. The highly motile cells produce a hydrodynamic flow field that drags surface-bound host antibodies against the swimming direction towards the site of localized endocytosis. This mechanism of antibody removal is only effective if the trypanosomes move directionally with a certain threshold velocity [5]. This speed is usually not reached in cell culture. Therefore, we assumed that trypanosome motion in the natural habitat should be faster than under artificial conditions. So far, the effect of the microenvironment on the motion of trypanosomes, or other cells for that matter, has not been considered adequately. Moreover, it does not even appear to be clear exactly how trypanosomes swim. For more than 150 years, the parasites have been observed microscopically [30], [31], and their motion became accepted to be corkscrew-like. Thus, an alternative model of trypanosome swimming came as a surprise, in which the cells are driven by the progression of kinks along the cell body axis [14]. We have quantified in detail the biomechanics of trypanosome swimming, and could not confirm the proposed rocking-type of motion. By using high-speed and high-resolution fluorescence microscopy we show that the free segment of the attached flagellum generates a major part of the force required for directional movement. Since the cell is force and torque free at low Reynolds numbers, the generation of locomotive force by the flagellum is well described by the simple resistive force theory [32]. The force generated by each beat causes immediate translocation as “inertia plays no role whatsoever” [22], [23]. The translocation results in directional laminar flow around the trypanosome cell body. The attached part of the flagellum largely contributes to the cellular asymmetry and chirality, which causes the moving trypanosome to rotate, as any chiral body will be forced to rotate in a laminar flow field. As has been shown by Shaevitz *et al.* for *Spiroplasma* [33], a bihelical propulsion mechanism requires a periodic change of cellular chirality [34]. In other words, for trypanosomes to move in such a way the handedness of the cell body would have to change over time. This is certainly not the case, as we have analyzed several hundred 3D-fluorescence image data sets without identifying a single trypanosome cell with clockwise chirality (Fig. 3*A*).

The here proposed complex mechanism of trypanosome movement was confirmed by conducting elaborate computer simulations with size parameters defined by the experiments.

The sinusoidal two-dimensional beat of the flagellum was applied to a trypanosome cell surface model, established on the basis of three-dimensional microscopic data. The cell model reacted to the flow field created by its motion and moved as observed in the experiments, rotating and describing a helical path. This analysis underlines the predictive and confirmatory power of computer simulations for analyzing complex biological traits such as cell motility.

The overall picture of a moving trypanosome indeed resembles a corkscrew (Fig. 3*B, C*), however, the flagellar tip itself produces a two-dimensional beat (Fig. 5). We conclude that the term “plane-rotational” would better describe the complex mode of trypanosome locomotion, even though this term may appear paradoxical at first glance.

In order to investigate how the trypanosomes plane-rotational type of motility performs under conditions of crowding and confinement, we trapped the parasites in arrays of micropillars. The leading flagellum efficiently pulls the trypanosome through the obstacle matrix, the mechanical interaction between cell body and pillars accelerating the cells to significantly higher velocities. This speed equals that in blood and is sufficient for the hydrodynamic flow-induced antibody clearance in the host. Thus, trypanosomes exploit friction forces for efficient locomotion. These forces were surprisingly also seen to act in the reversal of the trypanosomes swimming direction. Unidirectional tip-to-base beats effectively propel the cell forwards, while interspersed base-to-tip beats lead to a change of swimming direction. Continued interference of base-to-tip beats causes cells to tumble, but in the presence of mechanical resistance, successive, uninterrupted base-to-tip beating can be sustained, which directly causes the trypanosomes to swim backwards, also reversing their rotational movement. Thus, the parasite exhibits versatile three-dimensional movement capabilities, directly exploiting the physical nature of its surroundings. These can easily be envisaged to be of avail for the trypanosomes movement through tight spaces, as the parasite has to be maneuvered through tissue spaces and has to traverse the blood-brain-barrier. The environmentally induced reversal of swimming direction is a foolproof way of avoiding dead ends.

The fact that motility contributes to survival in the host and hence represents a virulence factor, points to strong selective pressure acting on the blood parasite. The plane-rotational trypanosome motility can be regarded as a genetically fixed adaptation to the crowded environment in the host. However, trypanosome motion is not an easily traceable quantitative trait, since not only the flagellum is causative for locomotion but also the shape and architecture of the entire cell. The half-turn attachment of the flagellum with its unique tip-to-base beat, the elastic paraflagellar rod, as well as the flexible, cage-like cytoskeleton jointly contribute to the complex motion pattern. Thus, one could argue that evolution has shaped the trypanosome cell for expedient locomotion in blood. This, however, is not entirely true, since throughout its complex life cycle the parasite experiences dramatically different micro-environmental conditions. In mammals, trypanosomes not only thrive in blood, but also swim in tissue spaces, in lymph and even in cerebrospinal fluid. Obviously, the flow regime and the degree of crowding in these areas vary greatly. Furthermore, the parasites have to leave the mammal with the blood meal of the tsetse fly. A series of developmental stage transitions takes place in distinct parts of the insect body. In order to survive the tour de force through the fly, the parasites must adjust their mode of motility.

To study the cooperative action of molecular and environmental cues controlling the motion pattern during trypanosome development seems a rewarding task. The unusually homogeneous cell surface architecture and the ease of genetic manipulation render African trypanosomes ideal objects for studies at the crossroads of micro-/nanoflow physics, membrane biochemistry and genetics. Lastly, understanding the microenvironment-dependence of trypanosome motility could offer unforeseen ways of combatting one of the most neglected tropical diseases, the African sleeping sickness [35].

## Materials and Methods

### Cells

Wildtype bloodstream form (BSF) *Trypanosoma brucei brucei*, strain 427 [36], Molteno Institute Trypanozoon antigen type 1.6, were cultivated in suspension at 37°C, 5% CO_2_ in HMI-9 medium, including a final volume of 10% FCS (Sigma-Aldrich, Taufkirchen, Germany). Cells were kept in the exponential growth phase at a density less than 5×10^5^ cells/ml by dilution with fresh culture medium.

Bovine blood cells were washed in isotonic saline solution (0.9% w/v NaCl) by centrifugation (3000 g, 5 min, 4°C) and resuspended in FCS to a final volume of 45% v/v.

### Surface staining

Live cells were surface-stained with 1 mM of AMCA-sulfo-NHS (Pierce, Rockford, IL) or Atto488-NHS (Atto-Tec, Siegen, Germany) for 10 min, immediately before each experiment. The incubation was carried out on ice and cells were kept in the dark. Unbound dye was removed by washing twice with ice-cold TDB at 2000×g for 90 s.

### Microscopy

All images were acquired with a fully automated fluorescence microscope iMIC (TILL Photonics, Gräfelfing, Germany), controlled by our own software (written in C/C++ and Java; Heddergott et al., in preparation) and equipped with 100×(NA 1.4) and 60×(1.45 NA) objectives (Olympus). Images were recorded with the CCD cameras pco.1600 or sensicam.qe (PCO AG, Kelheim, Germany).

For high-speed light microscopy, a Phantom v9.1 camera (Vision Research, Wayne, NJ) was used and xyt-image series were acquired at 200–1000 fps. A frame-rate of 200 fps was found to be sufficient for quantitative analysis of the fast flagellar beat of trypanosomes. At this rate the minimum required sampling frequency with regard to the Nyquist-Shannon sampling theorem was over-fulfilled 5-times.

For high-speed fluorescence microscopy, a beta-version of the sCMOS camera pco.edge (PCO) was used at frame-rates of 200–400 fps.

For 3D-modeling of fixed cells, xyz-stacks were acquired in 100 nm steps. The cells were fixed in a final concentration of 4% w/v formaldehyde and 0.25% v/v glutaraldehyde in 0.1 M HEPES buffer over night at 4°C. The stacks were deconvolved using Huygens Essential software (version 3.7.0, SVI, Hilversum, Netherlands). 3D maximum intensity projection volume models were generated from these stacks, an edge detection filter (Sobel) was applied and the model was false-colored in Amira (version 5.2.2, Visage Imaging, Berlin, Germany). Animations of 3D-models and annotated Videos were produced with Amira or Imaris (version 7.2.1, Bitplane, Zurich, Switzerland). Flagella were traced and their length measured in Amira.

Cells were imaged in a two-dimensional setup of ∼10 µm height between a microscopic slide and a 24×60 mm coverslip, or in free suspension in translucent channels of 200–800 µm height (ibidi GmbH, Martinsried, Germany), allowing unrestricted motion of trypanosomes in three dimensions.

### PDMS-Pillars

We used regularly aligned arrays of chemically inert poly-dimethyl siloxane (PDMS) pillars consisting of at least several hundred single pillars distributed across an area of more than 1 mm^2^. The pillars had a height of about 20 µm and were covered by a gas permeable film (lumox, Greiner Bio-One GmbH, Frickenhausen, Germany). Arrays of different combinations of spacing (3–20 µm) and diameter (5–12 µm) were used. Cells were applied in a small volume of about 10 µl of TDB (trypanosome dilution buffer: 20 mM Na_2_HPO_4_, 2 mM NaH_2_PO_4_, pH 7.7, 20 mM glucose, 5 mM KCl, 80 mM NaCl, 1 mM MgSO_4_) onto the pillar array. The *in vitr*o setup was chosen to exclude any undefined chemical cues. Only cells in the focus plane, set to the center of the pillar height, were included in the analysis.

### Generation of trajectories

Xyt-image series were acquired for at least 30 seconds. From these, the position of individual cells was determined by center-of-mass calculations. Velocities were measured as the sum of Euclidian distances between the single points of these trajectories. All analyses were done using our own software written in Java (Heddergott *et al.*, in preparation).

### Time-dependent tomography

Xyt-image series of directionally swimming trypanosomes were acquired at frame rates of 500–1000 Hz. Successive flagellar beats were analyzed and one image depicting the beginning of each beat was selected. The two-dimensional view of these images was compared and identical periodically repeating phases were identified. For example, in Fig. 4, after six flagellar beats the cell had returned to its original spatial orientation. The images selected from this period of movement were used for the tomography, using the 3 ds Max software (Autodesk Inc., San Rafael, CA). The cell contours were traced in each image and extruded to a three-dimensional object. These 3D-representations of successive beats were then aligned to an anterior-posterior axis. The 3D-models were rotated around this axis by a constant angle per beat and the intersecting regions of the rotated models were calculated and extracted to produce a tomographic 3D-model of the original object. A correct three-dimensional model of a trypanosome cell body was only produced when the rotational angle per beat was in the range of (50°±10°) and the rotation was unidirectional.

### Triangulated surface model of a trypanosome

In order to simulate the spindle-shaped trypanosome cell body we constructed a model cell body using vertices connected by elastic springs and also implemented a bending rigidity with the help of an appropriate bending potential [29]. In detail, we created 20 circles which were spaced with a unit distance along the long axis of the cell body. The body's radius is given by equation (1).
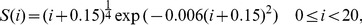
(1)
Equation (1) thus defines the thickness of the cell body. As shown in Fig. 6*A*, along each circle created by equation (1), 10 vertices are equally distributed with an angular displacement of π/5 radians.

Each circle vertex was connected to the corresponding vertex of an adjacent circle by Hookean springs shown in Fig. 6*A* as the bond vector between the points A and B. All 10 vertices distributed over one circle were connected to the adjacent circles, except the ones that constitute the beginning and the end of the body. The potential for the Hookean springs is given by:
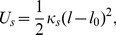
(2)where *κ_s_* is the spring constant and l_0_ is the equilibrium length of the springs. The integrity of the cell body was ensured by interconnecting all 10 vertices of each circle by springs shown in Fig. 6*A* as bonds between the points B, C and between the points B, D. In order to keep the cell body stable, relatively stiff springs were used throughout the simulations, with the spring constant *κ_s_* = 10^7^ in appropriate reduced units.

A bending potential was applied along the dashed line shown in Figure 6*B* (a) of the cell body. In order to apply the bending potential, the 10 lines which make up the long axis of the cell body were discretized as shown in Figure 6*B* (b). The bending potential that we applied is given by
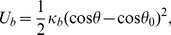
(3)where *θ* is the angle enclosed by the tangent vectors t*_i_* and t*_i+1_* as shown in 6*B* (c), *θ*_0_ is the equilibrium value of the angle and *κ_b_* is the bending stiffness. This configuration mimics the microtubule system of the trypanosome, which runs along the long axis of the cell body. Most microtubules originate at the posterior end of the cell body but do not always extend to the complete length of the cell. This makes the anterior end of the cell body more flexible than the posterior end. To account for this property of the cell body in our model, the bending stiffness was reduced in steps towards the anterior end by a factor of 0.95 starting from the center of the cell body. The resulting bending stiffness at the end of the cell body is (0.95)^10^ *κ_b_* = 0.6*κ_b_*.

The flagellum was attached to the cell body so that it runs straight from the posterior end up to the center of the cell body and then performs a rotation of π radians around the cell body as shown by the blue line in Fig. 6*C*. Through this flagellum a planar bending wave (in our case a sine wave) was passed, given by the potential [37]:
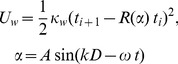
(4)where *κ_w_* is the strength of the bending wave and *R(α)* is a rotation matrix, which rotates a vector t*_i_* by an angle *α* about the local surface normal, *A* is the amplitude of the sine wave which is always kept as 1 in all the simulations, *D* is the distance from the posterior end of the flagellum to the point *i* on the flagellum, *k* = 2π/λ is the wave number, λ is the wavelength of the wave, *ω* is the angular frequency of the wave and *t* is the time in simulation units.

The open posterior and anterior ends were each closed by a semi-sphere of the respective diameter. For the trypanosome's cell body, the length to thickness ratio is about 25 µm/3.5 µm = 7.14. This ratio was closely matched in our model (7.32) and kept constant for all simulations reported in this work.

The velocities and positions of the vertices with mass *m* = 1 were always given in dimensionless units. They were updated during a molecular dynamics (MD) step by the velocity Verlet algorithm [38] using the time step *δt_MD_*,
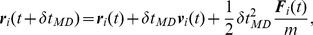
(5)

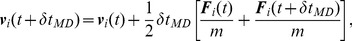
(6)Here ***r*** *_i_*, ***v*** *_i_* and 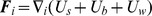 are the respective position, velocity, and force of the *i*th vertex of the cell body. To perform the gradient 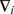 of the spring, bending and bending wave energy U*_s_*+U*_b_*+U*_w_* with respect to r*_i_*, the energies were discretized in the position variables r*_i_*. In order to maintain a stable structure we had to keep *δt_MD_*≪1, so in our simulation we always kept *δt_MD_* = 10^−4^.

### Simulation of fluid flow by the method of multi-particle collision dynamics

The method of multi-particle dynamics [24]–[28] has been widely used to study fluid dynamic problems such as the sedimentation of colloids [39], star polymers under Couette flow [40], and the thermal diffusion of a semi-flexible sheet [41], just to name a few examples. Biologically relevant systems have been addressed also, such as fluid vesicles under shear flow [42], [43], the dynamics of model swimmers called squirmers [44], [45], and swimming sperm cells both in two [37] and three [46] dimensions.

In multiple particle collision dynamics (MPCD) the fictitious fluid particles (point particles) are distributed in a 3-dimensional simulation box *L_box_* with periodic boundary conditions. In Fig. 6D we show the average flow field generated by the model trypanosome during swimming in the MPCD fluid. We employed a MPCD version using the Anderson thermostat with angular momentum conservation [38]. We started with a thermally equilibrated fluid by choosing the velocity of each particle from a three-dimensional Gaussian distribution with variance 3*k_B_T/m*, where T is the temperature of the fluid and m the mass of the fluid particle.

MPCD is divided into 2 different steps, one is called the streaming step and the other is called the collision step. In the streaming step the fluid particles are moved ballistically for a time δt.
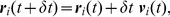
(7)Before each MPCD step we performed *p* MD steps of the cell body, where *p = δt/δt_MD_*. As a result of the MD steps and the following streaming step, fluid particles entered the cell body. All the fluid particles inside the cell body were tracked back to the surface of the cell body. Then each fluid particle was given the velocity of the nearest cell body vertex and moved for half the corresponding time step [47], thereby implementing the no-slip boundary condition. This procedure was continued until all particles were located outside the cell body. The total momentum was conserved during all these collisions with the cell body, taking into account its center-of-mass velocity. In our simulations we always kept *δt*≤0.01.

The streaming step is followed by the collision step. In the collision step we divided the simulation box into cells of length *a* = 1 and in each cell we changed the velocity *v_i_* of the fluid particle according to the following collision rule, which conserves both linear and angular momentum of the cell:
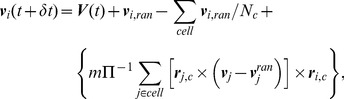
(8)Here the components of ***v*** *_i,ran_* are Gaussian random numbers, *N_c_* is the number of particles in the cell and ***V*** *(t)* is the center of mass velocity of all particles in the cell. To conserve angular momentum the last term is added, where Π is the moment of inertia tensor of the particles in the cell, and ***r***_i,c_ = ***r***_i_−***R***_c_ is the relative position of the particle *i* in the cell with respect to the center-of-mass mass position R*_c_* of all particles in the cell. The collision also included the vertices of the cell body. Since *δt*≪1, artificial correlations developed between particles of each cell [48], [49]. To avoid them we performed a random shift of the cells before each collision step. In our simulation time was measured in units of 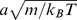.

The advantage of the collision rule is that it also acts as a thermostat, which is very important in our case as the cell body is always pumping in energy into our system with its constant beating. One can show that this sequence of streaming and collision steps is equivalent to solving the Navier-Stokes equations. MPCD provides an analytic expression for the viscosity, which depends on the number of particles per cell. In our simulations, we always chose the number of particles per cell as *ρ* = 10. The viscosity in the simulations is then given by the streaming (*η_st_* = 0.64*δt*) and collision (*η_cl_* = 0.036/*δt*) contributions and the total viscosity is *η* = *η_st_*+*η_cl_*. [50].

We tested our program code for two cases:

First, we determined the diffusion coefficient *D_sim_* of the passive cell body by monitoring its mean square displacement. We fitted the line 6 *D_sim_* t to the data, where for D*_sim_* we took the diffusion constant *D* of a cylinder whose radius is the mean radius of the cell body. The agreement is good (Fig. S1 *A*).

Second, we determined the Stokes friction coefficient γ of a sphere of radius *a* = 4 by dragging it through the fluid (viscosity 3.6) with various forces *F*, which we quantified by the Peclet number Pe. We obtained very good agreement with the exact value γ_0_ (Fig. S1 *B*).

A detailed numerical analysis of the swimming trypanosome cell body in its viscous environment using the MPCD method is presented in [29]. Here, we formulate an estimate for the swimming speed *v* of our model trypanosome. In [29] we found that *v*/*Lω* scales with the sperm number, where *L* is the length of the cell body and *ω* the angular frequency of the wave propagating along the flagellum. The sperm number is defined as
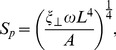
(9)where 
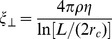
 is the friction coefficient of a cylinder of length *L* and radius *r_c_*, which is the mean radius of the trypanosome cell body, and *A* is the bending rigidity of the body. The sperm number compares frictional to bending forces. In the present work we used *δ*t = 0.01 which corresponds to a viscosity of 3.6 in MPCD units or in real units to a viscosity a factor of 50 smaller than in water. On the other hand, the angular frequency was *ω* = 2π*0.1 in MPCD units or 2π*330 Hz which is about a factor of 18 larger than the angular beat frequency of 2π*18.3±2.5 Hz reported in the experimental work. This means that instead of *S_p_* = 0.9 in our simulations we have a sperm number *S_p_* = 1.17, when real values are used. From the inset of Fig. 8 in [29] we find for this sperm number *v*/*Lω* = 6*10^−4^ or *v* = 1.7 µm/s. Thus, the swimming speed is smaller than the typical experimentally measured speed of 5 µm/s by a factor of three. We stress that the detailed values depend on a careful tuning of the cell body parameters. Our main goal was to contribute to understanding the swimming strategy of the African trypanosome. To this end, so far we have only modeled cells with a fully developed flagellum attached to the cell body. The analysis of different cell cycle stages will be part of future work.

### Ethics statement

Mice infection experiments are not regarded as animal experiments but as animal usage. The animal protocol used for infection of mice with trypanosomes adhered to German law (Tierschutzgesetz §4 Abs. 3). The experiments were approved by the appropriate German authorities (Regierung von Oberbayern, Az 211–2531.4).

## Supporting Information

Figure S1 Validation of the MPCD program code. (A) The diffusion coefficient *D_sim_* of the passive cell body was determined by monitoring its mean square displacement. We fitted the line 6 *D_sim_t* to the data, where for *D_sim_* we took the diffusion constant *D* of a cylinder with a radius equal to the mean radius of the cell body. The agreement is very good. (B) We determined the Stokes friction coefficient γ of a sphere with a radius of *a* = 4 by dragging it through the fluid (viscosity 3.6) with various forces *F*, which were quantified by the Peclet number Pe. There is very good agreement with the exact value γ_0_ as illustrated in the graph.
(TIFF)

Video S1 Particle displacement by trypanosomes. Beads of varying size and mass were used to simulate different degrees of particle crowding and to estimate the force a trypanosome flagellum can exert on the environment. (A, B) At low particle density and without stirring Dynabeads cluster similar to blood cells. Note that both the tumbling cell as well as the directionally swimming trypanosome is able to efficiently displace the beads. (C) Trypanosomes move effectively even when covered with a dense layer of beads. The inset shows displacement of large polystyrene beads recorded by high-speed microscopy. The mass of this particular type of beads (density: 1,05 g/cm^3^) and the average distance of displacement (roughly 2 µm in 30 ms) points to a force of minimally 5 pN produced by a flagellar deflection. (D) Left video: Fluorescently labeled trypanosomes recorded in freshly drawn mouse blood. The blood cells are displaced but not deformed by the trypanosomes. Right video: Trypanosomes displace erythrocytes in a 40 µm microfluidic channel without any obvious deformation of the cells. This means that the effective force of the trypanosome flagellum must be significantly smaller than 100 pN.
(WMV)

Video S2 (A) The path of the flagellum around the trypanosome cell body in three-dimensional representations. Trypanosomes were fluorescently surface-labeled with AMCA-sulfo-NHS, fixed and recorded as wide-field xyz stacks. The original and deconvolved stacks are shown. (B) The 3 d volume models generated from the deconvolved stacks are animated in a 180° turn and show the pathway of the flagellum wrapped approximately a half turn around the cell body. Using the volume models, the complete flagellum was traced and measured in three-dimensional space. The 180° turn relative to the body was registered on average along the first 11 µm of the flagellum, as measured from the emergence point at the posterior flagellar pocket, defined as the first anchor point, to the point on the opposite side of the cell body where the flagellum stopped wrapping around the body surface, defined as the second anchor point. The second third of the flagellum's length (full length ∼30 µm) followed the convex surface of the cell body. For clarity, the last free third of the flagellum is only shown for one cell.
(WMV)

Video S3 Stereo-view of fluorescent three-dimensional volume models. The rotating animations of the volume models in Video S2 are shown in stereographic view (red/cyan). Note that, due to missing depth cues, the non-stereographic animations can in some cases be perceived as rotating in the wrong direction, flipping the view of the flagellum-path. (For this so-called “silhouette illusion” effect see: Troje NF, McAdam M, 2010, “The viewing-from-above bias and the silhouette illusion” *i-Perception* **1**(3) 143–148).
(WMV)

Video S4 (A; B) High-speed fluorescence microscopic videos of surface-labeled trypanosomes. The flagellum can readily be traced in relation to the moving cell body, clearly revealing a rotational movement, as the flagellum periodically appears on one side of the body and disappears on the other side. (C) The trace of the fluorescently labeled cell shown in Fig. 3*B* is a projection view of this high-speed fluorescence data set. (D) Overlay of the time-lapse data and the projection view reveal rotational type of trypanosome motion. The diameter and the amplitude of the path are presented. (E) The relationship of the amplitude of the swimming path to the dimension of red blood cells is illustrated by the schematic illustration, in which the erythrocytes are drawn to scale. Note that the average distance of blood cells is below 5 µm.
(WMV)

Video S5 High-speed transmitted light videos of persistently swimming trypanosomes. The original recordings at 200, 500 or 1000 fps are shown in real time or slowed down 5x–50x. The elapsed time in milliseconds is shown in the upper left corner. (A) Trypanosome motion on a glass slide in medium with blood viscosity. After 1300 milliseconds, this trypanosome transiently slows down. Following some beat reversals the cell continues to move with similar speed but a different swimming direction. (B) The swimming behavior of trypanosomes in wide microchannels is not fundamentally different from the conditions on glass slides. The planar beat of the free end of the flagellum becomes especially evident in cells that swim along the optical z-axis and thus appear from the depth of the channel. (C) At low viscosity the swimming efficiency of trypanosomes is reduced. This results in decreased average velocity. Compare the 20-times slowed video with the comparable one in (B) to see the direct effect that increased viscosity has on swimming performance.
(WMV)

Video S6 High-speed transmitted light videos of persistently swimming trypanosomes. The original recordings at 200 fps are shown in real-time or slowed down 5× and 10×. (A) At low viscosity trypanosomes motility is inefficient and the flagellar beats appear irregular. (B) When the medium has blood viscosity the trypanosomes move more directional and the flagellar beats appear more regular.
(WMV)

Video S7 Z-axis-oscillation high-speed transmitted light microscopy of trypanosome motion. The swimming cell was recorded at 200 fps while the focal plane was periodically shifted up and down by 5 µm at a frequency of 50 Hz using a computer-controlled piezo Z-stepper (see inset showing a sketch of the object stage of the inverse iMIC microscope (TILL Photonics) used). Recordings are shown at real-time and slowed down 5× and 10×. Owing to the rotational motion of the cells they are never completely out of focus, although the cell diameter (max. 3–4 µm) is smaller than the 5 µm the objective lens is shifted 50-times per second.
(WMV)

Video S8 The planar beat of the free flagellum, wave progression along the cell and rotation of the cell body. A high-speed video that was analyzed frame by frame is shown in an annotated slow motion version of the original video (A) and in still images from this video (B). In both representations the single beats originating at the free anterior tip of the flagellum are shown. Additionally the distance of translocation corresponding to each beat is annotated. The still images show each half beat of the first six flagellar beats in the video. This corresponds to a rotation of 360° around the anterior-posterior axis, as can be seen by the identical orientation of the directionally swimming cell after these six beats (i.e. 0 ms and 364 ms). The graph in (A) shows the measurements of nucleus (blue) and posterior tip movement (red) in swimming direction as shown in the video below with a resolution of 10 ms. The time stretches corresponding to single sequential flagellar beats are superimposed in grey. Note that each flagellar beat produces a distinct propulsive force resulting in movement along the anterior-posterior axis. The velocity (force produced by the wave) in swimming direction varies as a function of (1) the planar wave's asymmetry as it runs into the cell body (force varies as a function of flagellar beat frequency) and (2) the helical movement of the cell perpendicular to the movement in swimming direction (net translocation) in y- and z-direction.
(WMV)

Video S9 Efficient locomotion of trypanosomes in PDMS pillar arrays. (A, B) Trypanosomes were fluorescently surface-labeled with AMCA-sulfo-NHS and observed in regular 4 µm-PDMS pillar arrays. High-speed videos were recorded using sCMOS technology (pco.edge camera). (C) Transmitted light microscopic example of trypanosomes swimming in arrays of pillars with two different sizes (10 and 12 µm). (D) Tracing fluorescently labeled trypanosomes in pillar arrays shows maximum swimming velocities of about 40 µm s^−1^. (E) Maximum intensity projection (left) and selected still images from a high-speed fluorescence (xyt) image series of a fluorescently labeled trypanosome swimming through an array of pillars (4 µm spacing), acquired at a frame-rate of 400 images per second. Note that the curvatures of the flagellum and the cell body closely reflect the shape of the pillars (positions indicated by “x”). Scale bar = 5 µm. (F) Illustration of the experimental setup.
(WMV)

Video S10 Out of focus information reveals three-dimensional information. (A) High speed transmitted light microscopic video corresponding to the still images in Fig. 3. The in and out of focus phases could be assigned in a periodic pattern to the projection of the cells path in order to show the helical nature of the path. In the slow motion video the rotation can be seen directly. Note the appearance and disappearance of the flagellum above or beneath the cell body, as it comes in and goes out of focus. (B) Volume-rendered models of trypanosomes, surface-labeled with AMCA-sulfo-NHS. The course of the flagellum attached to the cell body (closed arrows) is clearly visible from the flagellar pocket (open arrows) to the anterior free end. The flagellum characteristically describes a turn of about 180 degrees around the cell body, counter-clockwise in swimming direction. (C) In order to supplement the description of the three-dimensional helical path, the free anterior tip of the flagellum is traced. Note that the path of the flagellar tip along the time axis (blue) depicts a wave form that corresponds to the spatial orientation of the planar wave of the free part of the flagellum. The visible amplitude is highest when the wave lies in the viewer's xy-plane and lowest when it has rotated 90° and lies in the viewer's xz-plane.
(WMV)

Video S11 Trypanosomes are able to persistently swim backwards, confined by narrowly spaced obstacles. Analysis of a trypanosome swimming backwards through a microarray of pillars with a spacing of 4 µm. The directional movement is shown first in real time, then in slow motion, while a series of waves running from the posterior to the anterior end is traced and annotated. Simultaneously, the translocation of the posterior end is traced. Each movement is caused by one flagellar beat, observed when the corresponding wave reaches the anterior free part of the flagellum. The corresponding absolved movements and flagellar beats are shown in a three-dimensional overview at the end of the video. Here, all traced movements are depicted as continuous structures along the time-axis, which is rotated in order to visualize the progression of translocation in time from bottom left to top right of the image. The cell performs successive base-to-tip beats that result in a flagellar curvature producing characteristic hook conformations, which make the flagellum deviate from the movement-axis further than the sinus curves produced by forward motion. This results in more irregular translocations of the cell body, which is nevertheless confined to a straight path by the surrounding pillars.
(WMV)

Video S12 Trypanosomes change swimming direction by reversing the direction of flagellar beats. Annotated, slow motion video of a cell showing persistent forward motion with short pauses that are accompanied by a change of direction. The trajectory of a cell with the directions of movement is shown in the inset. Each produced tip-to-base and base-to-tip beat is annotated and the movement of the resulting wave along the cell body is followed. Forward and reverse waves can be seen superimposing. The cell is slowed down briefly, when a reverse wave reaches the free end of the flagellum. The cell comes to an immediate halt when the forward beats are stopped. A few successive reverse beats then cause the cell to reverse and change direction before the tip-to-base beats resume and the cell immediately begins forward movement on its new path.
(WMV)

Video S13 Trypanosomes tumble as a result of continuously counter propagating waves. Two slow motion videos of a tumbling trypanosome each, with every single tip-to-base and base-to-tip wave followed by annotation as in Video S12. The cells show little directional movement, as the effects of the counter propagating waves cancel each other out. The adverse rotational movements produced by the counter propagating waves contort, stretch and rotate the cells in three dimensions. The production of flagellar waves can intermittently come to a complete halt, with only the most anterior part of the flagellum showing any bending.
(WMV)

Video S14 Simulation of trypanosome motion using multi-particle collision dynamics and a triangulated cell surface model. (A) Figure of the surface model of a trypanosome with a flagellum following a 180° turn around the cell. The flagellar beats were simulated by applying sine waves to the flagellum in tip-to-base or base-to-tip direction. (B) The videos of the simulation results show a rotational movement in anterior direction for tip-to-base waves and (C) a rotational movement perpendicular to the axis of movement for the base-to-tip waves.
(WMV)
