# Supplementary figures and images for: Trypanosome Motion Represents an Adaptation to the Crowded Environment of the Vertebrate Bloodstream
Source: PLoS Pathog. 2012 Nov 15;8(11):e1003023. doi: 10.1371/journal.ppat.1003023 (PMC3499580; doi:10.1371/journal.ppat.1003023)

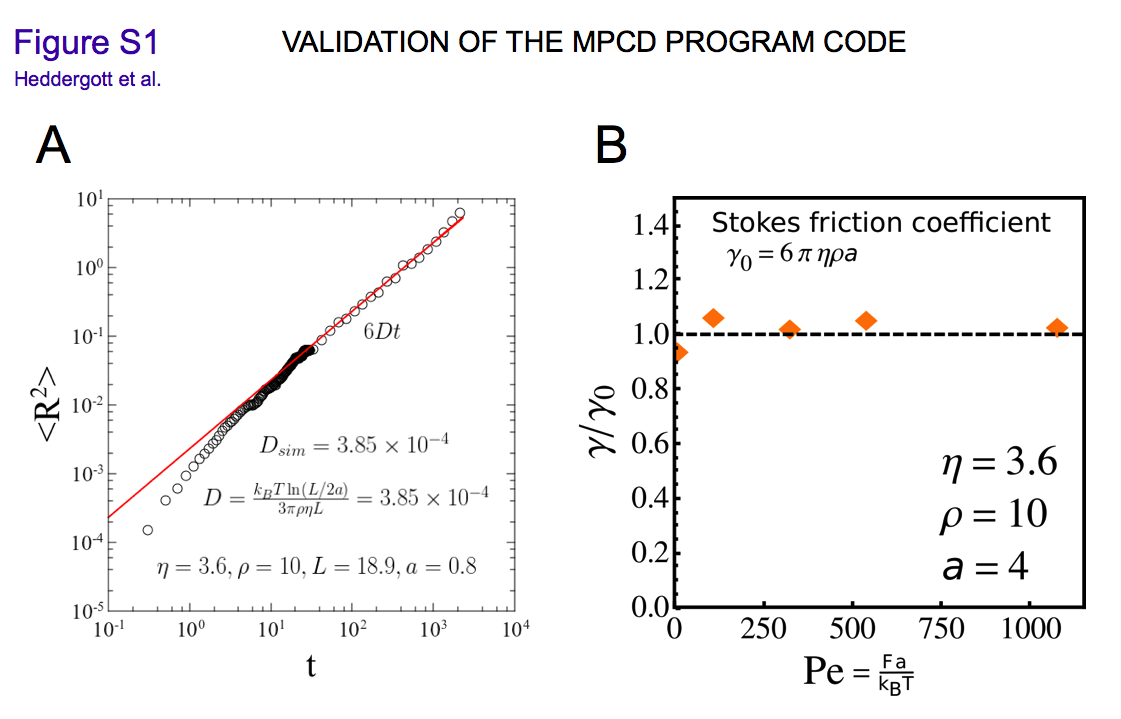

Supplement: Figure S1 — Validation of the MPCD program code. (A) The diffusion coefficient Dsim of the passive cell body was determined by monitoring its mean square displacement. We fitted the line 6 Dsimt to the data, where for Dsim we took the diffusion constant D of a cylinder with a radius equal to the mean radius of the cell body. The agreement is very good. (B) We determined the Stokes friction coefficient γ of a sphere with a radius of a = 4 by dragging it through the fluid (viscosity 3.6) with various forces F, which were quantified by the Peclet number Pe. There is very good agreement with the exact value γ0 as illustrated in the graph. (TIFF) [file ppat.1003023.s001.tif]
